# Monkeypox Virus Infection in 2 Female Travelers Returning to Vietnam from Dubai, United Arab Emirates, 2022

**DOI:** 10.3201/eid2904.221835

**Published:** 2023-04

**Authors:** Nguyen Thanh Dung, Le Manh Hung, Huynh Thi Thuy Hoa, Le Hong Nga, Nguyen Thi Thu Hong, Tang Chi Thuong, Nghiem My Ngoc, Nguyen Thi Han Ny, Vo Truong Quy, Vu Thi Kim Thoa, Nguyen Thi Thanh, Phan Vinh Tho, Le Mau Toan, Vo Minh Quang, Dinh Nguyen Huy Man, Nguyen Tan Phat, Tran Thi Lan Phuong, Tran Thi Thanh Tam, Phạm Thi Ngoc Thoa, Nguyen Hong Tam, Truong Thi Thanh Lan, Tran Tan Thanh, Sebastian Maurer-Stroh, Le Thuy Thuy Khanh, Lam Minh Yen, Nguyen Huu Hung, Guy Thwaites, Nguyen Le Nhu Tung, Louise Thwaites, Nguyen Van Vinh Chau, Nguyen To Anh, Le Van Tan

**Affiliations:** Hospital for Tropical Diseases, Ho Chi Minh City, Vietnam (N.T. Dung, L.M. Hung, H.T.T. Hoa, N.M. Ngoc, V.T. Quy, V.T.K. Thoa, N.T. Thanh, P.V. Tho, L.M. Toan, V.M. Quang, D.N.H. Man, N.T. Phat, T.T.L. Phuong, T.T.T. Tam, P.T.N. Thoa, N.L.N. Tung);; Center for Disease Control, Ho Chi Minh City (L.H. Nga, N.H. Tam, T.T.T. Lan);; Oxford University Clinical Research Unit, Ho Chi Minh City (N.T.T. Hong, N.T.H. Ny, T.T. Thanh, L.T.T. Khanh, L.M. Yen, G. Thwaites, L. Thwaites, N.T. Anh, L.V. Tan);; Department of Health, Ho Chi Minh City (T.C. Thuong, N.H. Hung, N.V.V. Chau);; Agency for Science, Technology and Research, Singapore (S. Maurer-Stroh);; University of Oxford, Oxford, England, UK (G. Thwaites, L. Thwaites, L.V. Tan)

**Keywords:** mpox, viruses, zoonoses, sexually transmitted infections, Vietnam, Dubai, United Arab Emirates, monkeypox virus

## Abstract

Mpox was diagnosed in 2 women returning to Vietnam from the United Arab Emirates. The monkeypox viruses belonged to an emerging sublineage, A.2.1, distinct from B.1, which is responsible for the ongoing multicountry outbreak. Women could contribute to mpox transmission, and enhanced genomic surveillance is needed to clarify pathogen evolution.

By January 12, 2023, more than 84,500 mpox cases and 80 deaths had been reported from 110 countries because of an ongoing multicountry outbreak (*1*). Cases from Europe and Americas accounted for >98% of reported cases, and only 35 cases had been reported from Southeast Asia (*1*). The outbreak has been characterized by involvement of networks of men who have sex with men; women have accounted for only 3.4% of 74,673 reported cases for which gender data were available (*1*). We report virologic, epidemiologic, and clinical features of mpox occurring in 2 women returning to Vietnam from travel to Dubai, United Arab Emirates.

## The Study

The case-patients were treated at the Hospital for Tropical Diseases (HTD) in Ho Chi Minh City, Vietnam. HTD is a tertiary referral infectious diseases hospital and the designated hospital for receiving and treating mpox patients in Ho Chi Minh City, which has a population of ≈10 million. The study was approved by the HTD Institutional Review Board (approval no. 1066/BVBND-HDDD) and Oxford Tropical Research Ethics Committee (approval no. 1023-13). The patients provided written informed consent for the study.

**Figure 1:**
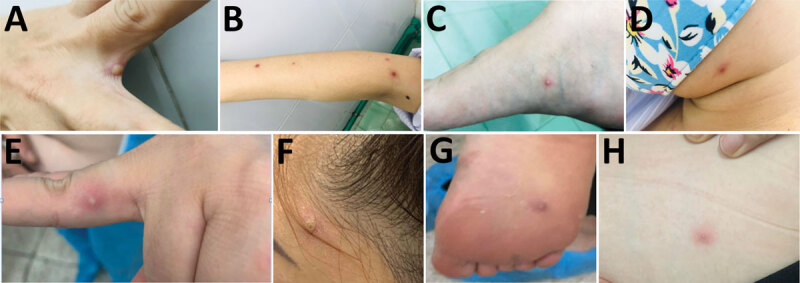
Monkeypox virus lesions from 2 female travelers returning to Vietnam from Dubai, United Arab Emirates, 2022. A–D) patient 1; E–H) patient 2. Images show lesions sporadically distributed on different body parts, including on patient 1 between 2 fingers (A), right arm (B), right foot (C), and face (D); and for patient 2, on a finger (E), the face (F), the arch of the right foot (G), and abdomen (H).

Patient 1 was a 35-year-old woman who was referred to HTD in September 2022. At admission, she had maculopapular lesions on various parts of her body (Figure 1, panels A–D), including the genital area (not shown). The patient had been in Dubai during July–September 2022 and had sexual contact with 2 male partners during her stay. The most recent contact was in mid-September; 5 days after the contact, she had fever, headache, chills, cough, sore throat, muscle pain, and tiredness, accompanied by a maculopapular rash in the genital area. Her symptoms resolved after 4 days except for the rash, and she returned to HCMC. Upon returning, additional lesions developed in her mouth and on her back and upper and lower limbs. No information on the clinical status or sexual orientation of her partners was available. Her admission lesion swab tested positive for monkeypox virus (MPXV) by LightMix Modular Monkeypox Virus Kit (TIB Molbiol, https://www.tib-molbiol.de) with a cycle threshold (Ct) value of 18.05 (Appendix Table 1) and for varicella zoster virus (VZV) by VZV Real-TM (Sacace Biotechnologies, https://sacace.com) with a Ct of 30.5.

Patient 2 was a 38-year-old woman who was a friend of patient 1. She reported that she was in Dubai during late September through mid-October 2022 and had a sexual encounter with a male partner at in early October. She noted that the partner had a small rash on his penis and mild fever on the day of the encounter. No information about the partner’s other sexual contacts is available. Nine days after the contact, she had fever, tiredness, and vomiting. Although her symptoms resolved after 4 days, a maculopapular rash started to develop on various parts of her body, including her face, a finger of the left hand, the arch of her right foot, and her abdomen (Figure 1, panels E–H). After consulting patient 1, patient 2 decided to fly back to Vietnam for treatment. Before departure she contacted Ho Chi Minh City Center for Disease Control for guidance on the isolation procedure at arrival. Patient 2 was transferred to HTD on arrival. She tested positive for MPXV by PCR at admission via LightMix Modular Monkeypox Virus Kit with a Ct value of 19.40.

At admission to HTD, both patients were afebrile. Except for an elevated alanine aminotransferase in patient 2, all blood test results were unremarkable (Appendix Table 2). All vital signs during hospitalization were measured by using wearable devices (Appendix Figure), as part of an observational study to enable remote patient monitoring (*2*), and measurements were within reference limits (data not shown). Test results for HIV and syphilis were negative. Because of the VZV co-infection, supporting a recent report (*3*), patient 1 was given oral acyclovir (800 mg 5×/d for 5 d). No other specific treatments were given. The patients were isolated, according to local health regulations, and their conditions remained stable without complications. After all lesions were completely healed, they were discharged.

**Figure 2:**
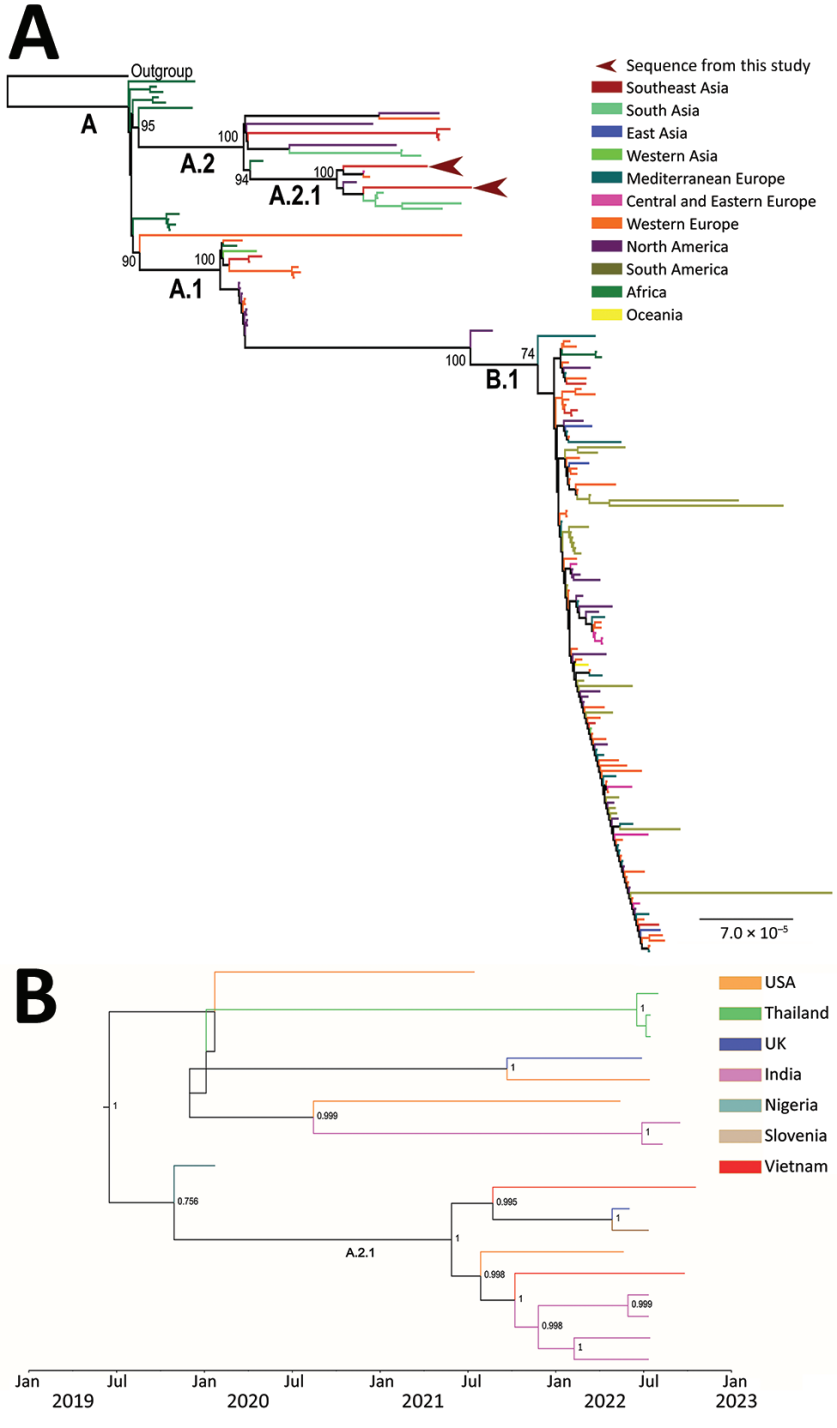
Phylogenetic tree of monkeypox virus infection in 2 female travelers returning to Vietnam from Dubai, United Arab Emirates, 2022. A) Maximum-likelihood phylogenetic tree illustrating the relatedness between virus sequences obtained in this study (Genbank accession nos. OP936000 and OP936001) and reference strains. Most sequences of sublineage A.2.1 from South Asia belong to a cluster from India (green) reported in July 2022 from persons with a travel history to the United Arab Emirates. The remaining sequences of A.2.1 from outside Asia included 1 isolated in the United Kingdom in June 2022, 1 isolated in the United States in May 2022, and 1 isolated in Nigeria in January 2020. Scale bar indicates nucleotide substitutions per site. B) Maximum-clade credibility tree of monkeypox virus lineage A.2. Red branches are sequences from this study.

To characterize the virus, we used metagenomics to obtain whole-genome sequences from the admission swab sample from patient 2 and a lesion swab sample with Ct value of 18.19 collected from patient 1 during follow-up (*4*,*5*) (Appendix Table 1). We obtained 2 nearly complete MPXV genomes with coverage of 97.7% from patient 1 and 95% from patient 2. We determined viral lineage by using NextClade (*6*). Phylogenetic analysis suggested the sequences belonged to clade IIb, sublineage A.2.1 (Figure 2). Both sequences carried defined mutations of sublineage A.2.1, including C25072T, A140492C, and C179537T (*7*). In addition, we found a novel nonsynonymous substitution from threonine to isoleucine in amino 717 (T717I) of the polymerase protein in the sequence from patient 1. This mutation was not detected in any previously reported MPXV genomes. The estimated time to the most recent common ancestor of lineage A.2, including sublineage A.2.1, was May 27, 2019 (range August 3, 2018–January 23, 2020).

Additional samples were collected from patient 1 for PCR testing during follow up. Of those, 1 rectal (Ct value 33.30) and 1 pharyngeal lesion (Ct value 33.27) swab sample were also positive for MPXV by PCR (Appendix Table 1). Whole-genome sequencing of follow-up samples was hampered by low viral load (Appendix Table 1). We performed Sanger sequencing of a 531-bp PCR amplicon spanning the nonsynonymous mutation (Appendix Table 3), which confirmed presence of the T717I substitution in both the rectal and pharyngeal lesion swabs (data not shown), suggesting that this mutation was sampling-site independent (*8*).

MPXV consists of 2 main clades, I and II (*9*), and clade II includes subclades IIa and IIb. Clade I is endemic in Central Africa and clade IIa in West Africa. Clade IIb is responsible for the ongoing multicountry outbreak, and B.1 is the predominant virus lineage (*9*). In contrast to lineage B.1, sublineage A.2.1 of clade IIb has only recently been documented in a cluster of persons from India with a travel history to United Arab Emirates (*7*). In addition, 3 other A.2.1 sequences deposited to GISAID (https://www.gisaid.org) originated in the United Kingdom, the United States, and Nigeria (Figure 1). Because MPXV evolves slowly, the genetic difference between the 2 sequences in this study coupled with the long branches of the A.2.1 cluster on the phylogenetic tree point to the possibility of silent transmission. Alternatively, the current sampling approach might have failed to comprehensively capture the genetic diversity of circulating MPXV strains worldwide. Collectively, these data suggest that lineage A.2, including sublineage A.2.1, likely represents an emerging MPXV lineage, supported by the results of time-scale phylogenetic analysis. Thus, multiple MPXV lineages likely are circulating and causing the ongoing multicountry outbreak.

## Conclusions

We report 2 MPXV infections in women returning to Vietnam from Dubai, adding to the few reports of mpox in women (*10*,*11*). The viral strain belonged to sublineage A.2.1 and was phylogenetically distinct from sublineage B.1 circulating and causing the ongoing multicountry outbreak in Europe and Americas (*9*,*12*). Both patients had sexual contacts with male partners in Dubai, 5 and 9 days before symptoms developed. Our findings suggest that contribution of women in MPXV transmission networks might be greater than previously appreciated. Enhanced genomic surveillance is needed to clarify the epidemiology and evolution of MPXV.

Appendix Additional information on mpox virus infection in 2 female travelers returning to Vietnam from Dubai, United Arab Emirates, 2022.
