# Supplementary material for: Monkeypox Virus Infection in 2 Female Travelers Returning to Vietnam from Dubai, United Arab Emirates, 2022
Source: Emerg Infect Dis. 2023 Apr;29(4):778–81. doi: 10.3201/eid2904.221835 (PMC10045713; doi:10.3201/eid2904.221835)
Supplement: Appendix — Additional information on mpox virus infection in 2 female travelers returning to Vietnam from Dubai, United Arab Emirates, 2022. [file 22-1835-Techapp-s1.pdf]

Article DOI: https://doi.org/10.3201/eid2904.221835

Monkeypox Virus Infection in 2 Female Travelers Returning to Vietnam from Dubai, United Arab Emirates, 2022

Appendix

Material and Methods

Metagenomic Mehtods and Sequence Assembly

Metagenomics was carried out as previously described (*1*). In brief, to increase the chance of success obtaining the complete genome of the virus, before nucleic acid isolation, the PCR-positive lesion swab was subjected to a pretreatment step incorporating DNase and RNase to digest unwanted background. The pretreatment reaction included 100 µL of the lesion swab in viral transport medium, 20 U of turbo DNase and 2U RNase I (Ambion-Life Technology, ThermoFisher, https://www.thermofisher.com), and was incubated at 37°C for 30 min. The treated swab was then subjected to a viral DNA isolation step using DNeasy Blood & Tissue Kit (QIAGEN, https://www.qiagen.com), and finally separately recovered in 50 µL of the elution buffer provided with the extraction kit.

Then, 10 µL of the extracted DNA was converted to double stranded DNA by using a set of 96 non-ribosomal random primers (FR26RV-Endoh primers) (*1*), SuperScript III enzyme (Invitrogen, ThermoFisher Scientific, https://www.thermofisher.com), RNaseOUT (Invitrogen), exo-Klenow fragment (Ambion), and RNase H (Ambion). Subsequently, the synthesized dsDNA was randomly amplified using the FR20RV primer (5′-GCCGGAGCTCTGCAGATATC-3′). The obtained random PCR product was then subjected to library preparation steps by using COVIDSEQ Assay (Illumina, https://www.illumina.com), according to manufacturer's instructions. Prior to sequencing, the quantity and size of the prepared library was measured by using Qubit dsDNA HS kit (Invitrogen) and Agilent High Sensitivity D1000 kit (Aligent Technologies, https://www.agilent.com). 12 pM of the prepared library was sequenced by using MiSeq Reagent kit V3 (150 cycles) or MiSeq Nano reagent kit V2 (300 cycles) (Illumina) in a

MiSeq platform (Illumina). The sample was multiplexed and differentiated by double indexes using Nextera XT Index Kit (Illumina).

The raw sequence reads generated by Illumina MiSeq were assembled using a reference-based mapping strategy available in Geneiuos Prime 2022.2.2 (https://www.geneious.com), followed by manual editing of the obtained consensus. A monkeypox virus sequence (GISAID ID: EPI_ISL_13094227) was used as a reference.

In addition, to assess the sensitivity of the metagenomics assay in recovering the complete virus genome, a serial dilution series of the original swabs (10× and 100×) of patient 1 was prepared and sequenced using the procedure described above. The consensus sequences generated in this study were submitted to NCBI (GenBank)/GISAID and assigned accession numbers.

Phylogenetic Analysis

Pairwise alignment was performed using the tool available on Nextclade (*2*). Maximum likelihood phylogenetic tree was reconstructed using IQ Tree version 1.4.3 (*3*) applying Hasegawa Kishono Yano (HKY) nucleotide substitution model with invariant, and support for individual nodes was assessed by using a bootstrap procedure (1,000 replicates).

Time-Scale Phylogenetic Analysis

The analysis was carried on lineage A.2 sequences. The dataset consisted of 19 whole-genome sequences with available data about sampling dates. The temporal signal was assessed by using TempEst 1.5 (http://tree.bio.ed.ac.uk/software/tempest). Bayesian phylogenetic interference was carried in BEAST version 1.10.4 (http://beast.community) using HKY+Γ substitution model (as suggested by IQ Tree) under a strict clock model and constant size coalescent tree prior. A Bayesian Markov chain Monte Carlo framework (available in BEAST) was used with 50 million steps and sampling every 5,000 steps. We assessed convergence with a burn-in threshold of 10% using Tracer version 1.5 (http://tree.bio.ed.ac.uk/software/tracer). Maximum-clade credibility (MCC) trees were then summarized with TreeAnnotator (available in the BEAST package) and visualized in Figtree version 1.4.2 (http://tree.bio.ed.ac.uk/software/figtree).

References

1. Nguyen AT, Tran TT, Hoang VM, Nghiem NM, Le NN, Le TT, et al. Development and evaluation of a non-ribosomal random PCR and next-generation sequencing based assay for detection and sequencing of hand, foot and mouth disease pathogens. Virol J. 2016;13:125. PubMed https://doi.org/10.1186/s12985-016-0580-9

2. Aksamentov I, Roemer C, Hodcroft E, Neher R. Nextclade: clade assignment, mutation calling and quality control for viral genomes. J Open Source Softw. 2021;6:3773. https://doi.org/10.21105/joss.03773

3. Nguyen LT, Schmidt HA, von Haeseler A, Minh BQ. IQ-TREE: a fast and effective stochastic algorithm for estimating maximum-likelihood phylogenies. Mol Biol Evol. 2015;32:268–74. PubMed https://doi.org/10.1093/molbev/msu300

Appendix Table 1. PCR results for monkeypox virus infection in 2 female travelers returning to Vietnam from Dubai, United Arab Emirates, 2022*

Patient no.	Sample type	Sampling time point	Ct value
1	Lesion swab 1	At admission	18.05
1	Lesion swab 2	4 days after admission	18.19
1	Rectal swab	4 days after admission	33.30
1	Throat swab	4 days after admission	Negative
1	Vesicle swab in the mouth	5 days after admission	33.27
2	Lesion swab 1	At admission	19.4

*Ct, cycle threshold.

Appendix Table 2. Vital signs and blood tests of 2 female travelers with monkeypox virus infection after in returning to Vietnam from Dubai, United Arab Emirates, 2022*

Variables	Patient 1	Patient 2
Temperature	37°C	37°C
Heart rate, bpm	106	88
Respiratory rate, rpm	20	20
Blood pressure, mmHg	140/80	100/70
Leukocyte count, cells $\times 10^9$/L	9.37	11.96
Neutrophils, cells $\times 10^9$/L	3.73	4.17
Lymphocytes, cells $\times 10^9$/L	4.21	5.39
Monocytes, cells $\times 10^9$/L	0.49	0.51
Eosinophils, cells $\times 10^9$/L	0.34	0.66
Basophils, cells$\times 10^9$/L	0.09	0.19
Large unstained cells $\times 10^9$/L	0.51	1.04
Hemoglobin, g/dL	11.7	12.5
Mean corpuscular volume, fL	91	86.36
Mean corpuscular hemoglobin, pg	28.2	26.3
Platelet count, cells $\times 10^9$/L	336	289
Creatinine, µmol/L	55	44
Glucose, µmol/L	7.63	Not done
Aspartate aminotransferase, U/L	18	33
Alanine aminotransferase, U/L	28.7	70

*bpm, beats per minute; rpm, respirations per minute.

Appendix Table 3. Sequences of primers used to confirm the OPG071:T717I substitution from monkeypox virus infection in 2 female travelers returning to Vietnam from Dubai, United Arab Emirates, 2022*

Oligo name	Sequences	Length, bp
Mpox-T717I-F	5′-TCATCCGTCCTTCAGACAACA-3′	21
Mpox-T717I-R	5′-CGGCTAAGAGTTGCACATCC-3′	20

*T717I, threonine to isoleucine in amino 717

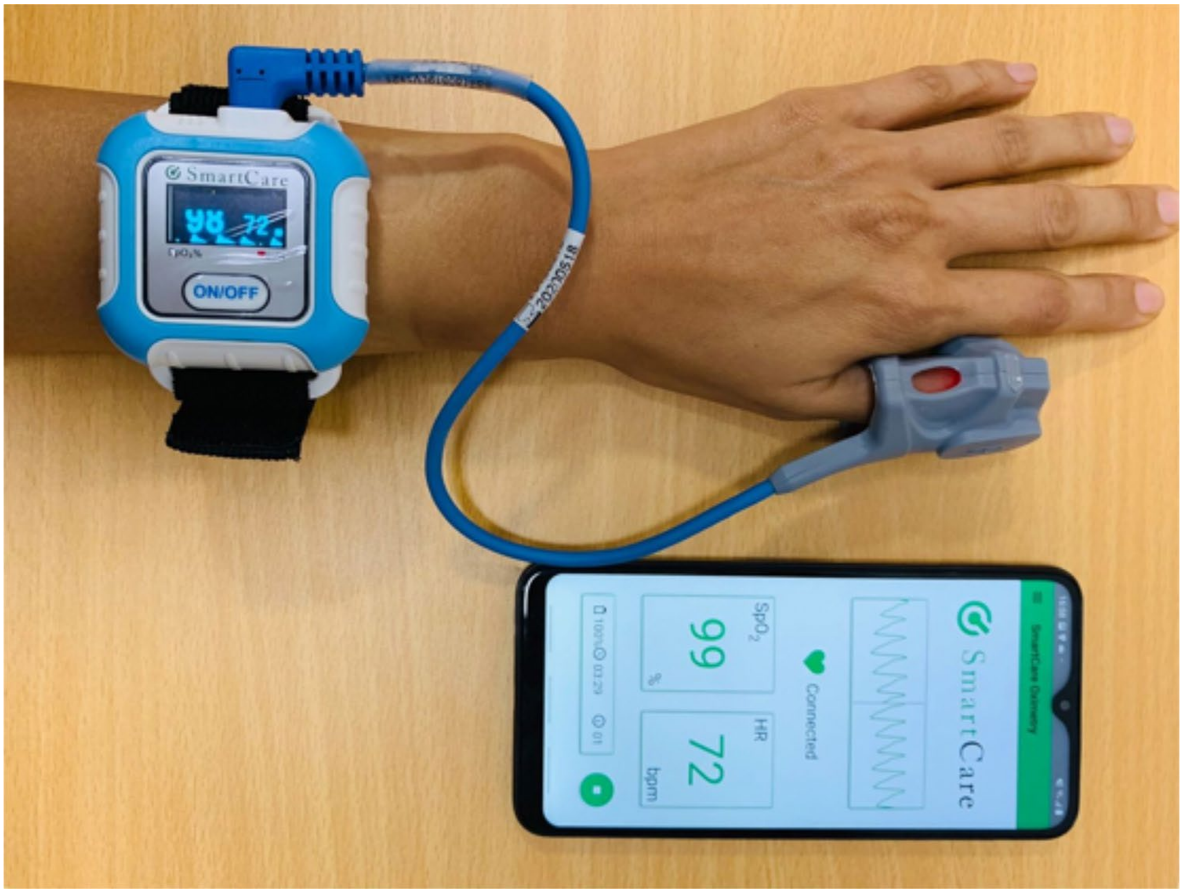

Appendix Figure. Wearable pulse oximeter device and smartphone display used for remote patient monitoring for monkeypox virus infection in 2 female travelers returning to Vietnam from Dubai, United Arab Emirates, 2022. The wearable device reduces clinical staff contact with patients.
